# Cortico‐cortical evoked potentials: Analytical techniques and emerging paradigms for epileptogenic zone localization

**DOI:** 10.1111/epi.18467

**Published:** 2025-05-23

**Authors:** Zekai Qiang, Jamie Norris, Gerald Cooray, Richard Rosch, Kai Miller, Dora Hermes, Aswin Chari, Martin Tisdall

**Affiliations:** ^1^ Developmental Neuroscience, Great Ormond Street Institute of Child Health University College London London UK; ^2^ Wellcome Centre for Human Neuroimaging University College London London UK; ^3^ Department of Neurophysiology Great Ormond Street Hospital London UK; ^4^ Department for Basic and Clinical Neuroscience, Institute of Psychiatry, Psychology and Neuroscience King's College London London UK; ^5^ Department of Physiology and Biomedical Engineering Mayo Clinic Rochester Minnesota USA; ^6^ Department of Neurologic Surgery Mayo Clinic Rochester Minnesota USA; ^7^ Department of Neurosurgery Great Ormond Street Hospital London UK

**Keywords:** CCEP, intracranial EEG, SEEG, SPES

## Abstract

Cortico‐cortical evoked potentials (CCEPs) are an active electrophysiological technique used during intracranial electroencephalography to evaluate the effective connectivity and influence of therapeutic stimulation between distinct cortical regions and pinpoint epileptogenic zones (EZs) in patients with epilepsy. Various methodologies have been implemented to analyze CCEPs and characterize the epileptogenic networks for EZ localization. Despite its promise, their interpretation remains challenging due to the large volumes of spatially and temporally complex data generated. Early studies focused largely on qualitative descriptors and predefined, semi‐quantitative features such as waveform morphology and peak latencies. However, these methods are limited by the significant heterogeneity in CCEP waveform conformations across patients and cortical regions. The specific technique used for extraction of features, such as the spectral band power and root mean squared values, remains open to empirical refinement, as does choice of appropriate latency windows, with no consensus reached regarding the optimal approach. Graph theoretical metrics such as degree centrality, betweenness centrality, and clustering coefficients can provide a rich representation of epileptogenic network connectivity. However, these metrics are often abstract and difficult to interpret in a clinical setting or to the non‐expert, and their neuroscientific substrates remains poorly understood. The lack of standardization in stimulation protocol and data‐processing pipelines has further contributed to inconsistency in reported findings. Emerging machine learning approaches have been increasingly applied to CCEP data, offering a more data‐driven and potentially generalizable way to identify electrophysiological biomarkers of the epileptogenic effective connectivity. In this article, we discuss qualitative, quantitative, and spectral features; network‐analytical metrics; and more recently, data driven methodologies aimed at improving the interpretability and clinical utility of CCEP data.

**Key points**

- Cortico‐cortical evoked potentials (CCEPs) are used during intracranial electroencephalography to evaluate effective connectivity between cortical regions, localize epileptogenic zones, and guide epilepsy surgery and neuromodulation approaches.
- CCEPs produce spatially and temporally complex data, with variability across patients and brain regions, making their interpretation and standardization difficult.
- Traditional methods focus on qualitative descriptors and semi‐quantitative features including spectral band power and latency‐amplitudes, but they lack consensus on optimal extraction techniques.
- Emerging solutions including machine learning offer a promising data‐driven approach to enhance biomarker identification and improve the interpretability and clinical utility of CCEP data.

## 1 INTRODUCTION

Approximately 25%–30% of individuals with epilepsy exhibit drug resistance (continued seizures despite an optimal antiseizure medication regimen), a condition that continues to result in significant morbidity and mortality.^1^, ^2^, ^3^ Comprehensive evaluations, including video–electroencephalography (EEG) telemetry and structural neuroimaging, have been critical for identifying suitable candidates for resective, disconnective, neuromodulatory, or ablative epilepsy surgery.^4^, ^5^, ^6^ However, reported success rates for these procedures remain highly variable in the literature.^7^ In cases where non‐invasive investigations yield inconclusive results, invasive techniques such as stereo‐electroencephalography (SEEG) or electrocorticography (ECoG), may provide additional information through high‐resolution examination of cortical activity. Despite these advanced approaches, the correlation between the extent of resected putative epileptogenic zone (EZ) and postoperative seizure freedom remains weak.^8^, ^9^ For example, in a cohort of pediatric patients who had 25–100% of seizure‐onset contacts, or presumed EZ resected, 68.2% achieved Engel Class I outcomes (seizure freedom) at a median follow‐up of 19.5 months.^8^ Notably, the definition of EZ has evolved since its inception in 1966, as brain regions identified as primary sources of epileptic seizure generation and organization through electroclinical correlations using SEEG recordings,^10^ to a more operationalized definition: the minimum amount of cortical tissue that must be resected, inactivated, or completely disconnected to achieve seizure freedom.^11^, ^12^ The latter scheme distinguishes the EZ—the cortical area which, if removed, could cure epilepsy—from related concepts such as the seizure‐onset zone, which merely indicates where seizure initiation is observed. Although these two regions may overlap, they do not always coincide. It is important to note that seizure‐freedom rates were lower in cases of extratemporal onset, reflecting the challenges of precisely defining the EZ in these regions, where pathological networks tend to be more diffuse and surgical boundaries less distinct.^8^, ^13^ Emerging evidence suggests that postoperative seizure outcomes may be influenced by broader network mechanisms beyond the observed seizure‐onset zones, emphasizing the need for dynamic approaches to investigate epileptogenic connectivity.^14^, ^15^

## 2 PRINCIPLES AND PRACTICE OF CCEPs

Cortico‐cortical evoked potentials (CCEPs) are an active electrophysiological technique used during SEEG and ECoG to infer directional connectivity between distinct cortical regions.^16^ SEEG and ECoG differ in their sensitivity to neural activity sources, which affects the interpretation of CCEP polarity. Due to the orientation of pyramidal cells toward ECoG electrodes, there are relatively consistent spatial relationships with neurophysiological sources, resulting in similar response types generating waveforms with consistent polarities. In contrast, SEEG electrodes penetrate tissue at various angles, creating variable relationships to neural sources. Consequently, identical neurophysiological activity may show very different CCEP waveform polarities depending on the angle from which it is sampled, leading to heterogeneous response patterns across anatomic locations.^17^ Although “CCEP” is the more widely used term for clarity, “single‐ pulse electrical stimulation” (SPES) is also used particularly when investigating epileptogenicity.^16^ Some authors define CCEPs exclusively as potentials evoked and measured from the cortical surface using strips or grid electrodes, but those recorded through SEEG as “pulse‐evoked potentials.”^18^ However, recent literature shows a convergence toward a procedure‐response distinction, characterizing SPES specifically as the stimulation method, whereas CCEP the resulting neurophysiological responses.^19^, ^20^ In this review, we follow this standardized framework where SPES denotes the action of delivering brief current pulses to specific brain sites, and CCEPs are the recorded responses that define effective connections between stimulus and response regions.^21^

**FIGURE 1:**
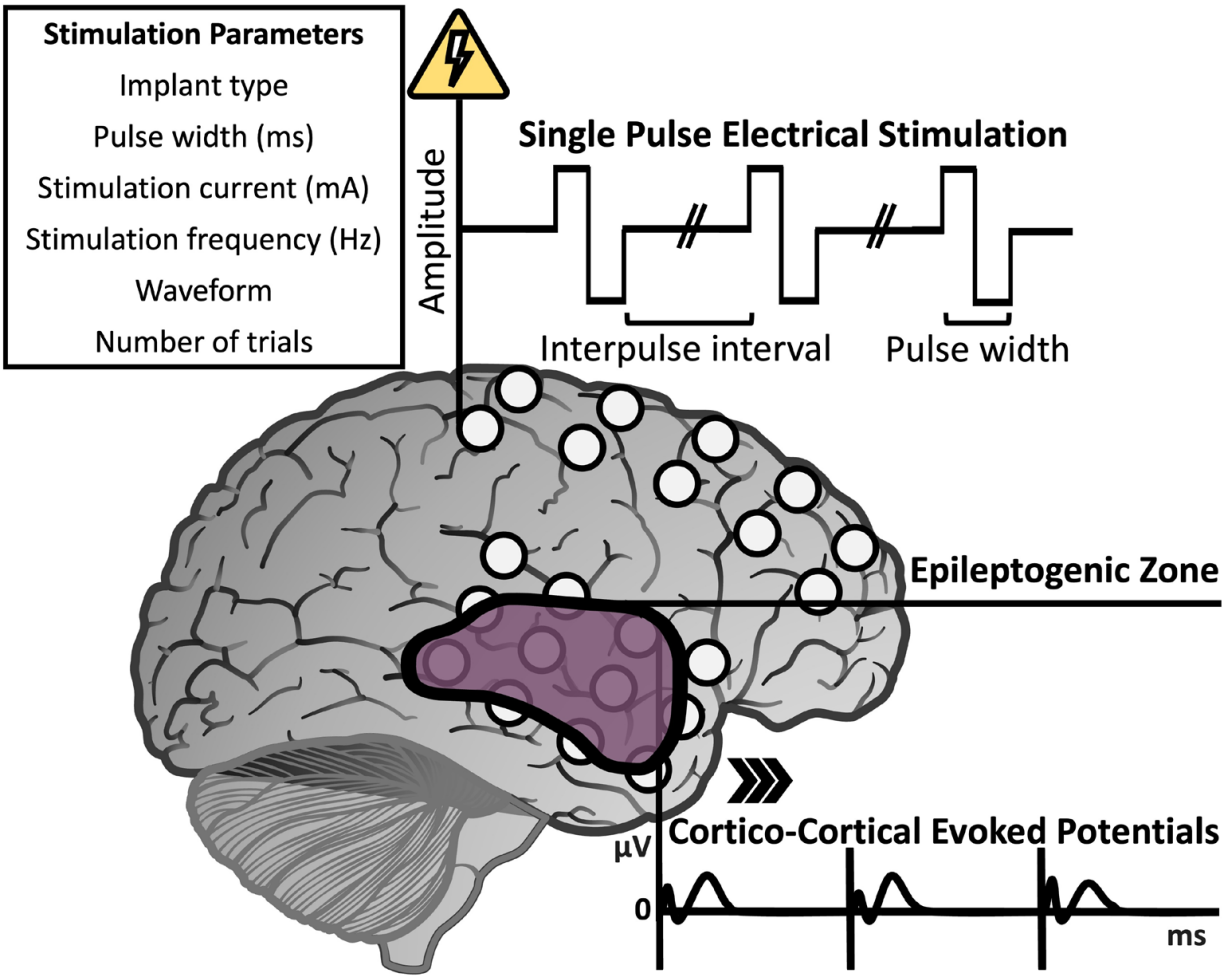
The single‐pulse electrical stimulation (SPES) procedure and the resultant cortico‐cortical evoked potential (CCEP) waveforms. The SPES protocol employs a variety of stimulation parameters, including pulse duration, frequency, and intensity. The elicited CCEPs are analyzed to localize the epileptogenic zones by identifying regions with distinct connectivity patterns and abnormal responses.

The generator mechanisms underlying CCEPs remain elusive. Using ECoG, SPES‐induced currents likely affect the neocortical surface through direct depolarization of pyramidal cell dendrites, activation of inhibitory interneurons, and stimulation of traversing axons, producing both orthodromic and antidromic action potentials.^22^ Although there is some antidromic contribution, signal propagation is thought to occur primarily through orthodromic cortico‐cortical and cortico‐subcortical–cortical projections.^23^, ^24^ At recording sites, pyramidal cells are postulated to generate field potentials characterized by early surface negativity followed by complex excitatory–inhibitory patterns across all cortical layers, over longer duration and lower frequency.^22^ Practically, the stimulation can be delivered either in a monopolar configuration using a distant extracranial electrode or in a bipolar configuration between adjacent electrode pairs within the brain (Figure 1). Optimal bipolar stimulation may involve 0.15 ms/phase pulses at 6–7 mA, which maximize early response amplitude and stabilize waveform morphology.^25^ In monopolar settings, stimulation of at least 5.5 mA yielded reliable responses.^26^ However, the charge per phase parameter may be the primary determinant of response magnitude, regardless of specific stimulation current combinations.^27^ Responses to monopolar stimuli may be more affected by volume conduction effects due to the larger net charge compared to bipolar.^28^ Volume conduction also impacts CCEP waveforms, especially their early components, and may account for most of the observed potential near the stimulation site.^29^

CCEPs are typically recorded after clinical seizures have been documented and baseline doses of antiepileptic drugs have been reinstated.^16^ However, a partial reduction in medication may sometimes be implemented to balance the risk of seizure induction against the benefit of observing more naturalistic connectivity patterns.^30^ The stimulation parameters used can vary across different studies and contribute to the resultant waveform morphologies (Table 1). The study of CCEPs has so far provided a unique opportunity to directly investigate human cortical networks in several regions, including the perisylvian, fronto‐parietal,^31^ hippocampo‐cingulate,^32^ and insular networks.^33^

**TABLE 1:** Summary of published CCEP studies.

Author, year	*N*	Age (years)	Implant type	Waveform	Analytical features	Main findings
Valentin 2002^34^	45	Mean: 33.8 Range: 14–58	ECoG, SEEG	Monophasic Pulsewidth: 0.3, 1 ms Current:1–8 mA Frequency: 0.1 Hz Trials: 10	Early responses, delayed responses	Areas investigated: temporal lobe. EZ markers: the distribution of delayed responses is strongly linked to seizure‐onset regions and may help identify areas of hyperexcitable cortex
Valentin 2005^35^	30	Median: 34 Range: 18–57	ECoG, SEEG	Monophasic Pulsewidth: 1 ms Current: 4–8 mA Frequency: 0.1–0.125 Hz Trials: 10	Early responses, delayed responses, repetitive responses	Areas investigated: frontal lobe EZ markers: stimulation of adjacent regions triggers delayed responses in epileptogenic areas, whereas frontal regions where stimulation evokes repetitive responses may be functionally abnormal
Valentin 2005^36^	40	Mean: 36 Range: 3–62	ECoG, SEEG	Monophasic Pulsewidth: 1 ms Current: 4–8 mA Frequency: 0.1–0.125 Hz Trials: 10	Early responses, delayed responses, repetitive responses	Areas investigated: temporal and frontal lobes Surgical outcomes are significantly better when areas with delayed or repetitive responses are fully resected; these regions also show structural abnormalities on neuropathological examination
Flanagan 2009^37^	35	Median: 14.2 Range: 0.75–17.6	ECoG, SEEG	Alternating monophasic Pulsewidth: 0.6–1 ms Current: 4–8 mA Frequency: 0.1–0.2 Hz Trials: 20	Early responses, stable responses, delayed responses, repetitive responses	Areas investigated: frontal, parietal, occipital, and temporal lobes Delayed or repetitive responses are present in 54% of patients and are associated with presumed epileptogenic lesions
Yu 2019^38^	11	Mean: 5.26 Range: 2.6–11	SEEG	Biphasic Pulsewidth: 0.3 ms Current: 10 mA Frequency: 1 Hz Trials: 15	Number of CCEPs where either N1 or N2 waves are present	Areas investigated: epileptogenic tuber, early‐stage propagating tuber, and perituberal cortexes Stimulation of epileptogenic tubers evoked significantly higher number of CCEPs in epileptogenic and perituberal tubers than stimulating non‐epileptogenic tubers
Cornblath 2023^39^	11	Mean: 37.8 Range: 21–55	SEEG	Biphasic Pulsewidth: 0.3 ms Current: 3 mA Frequency: 1 Hz Trials: 30	N1 and N2 amplitudes	Areas investigated: hippocampus and temporal structures EZ markers: N1 and N2 amplitudes are lower but more variable in the EZ N1 amplitudes synchronize with hippocampal low‐frequency oscillations, but did not localize epileptogenicity
Feys 2024^40^	20	Mean: 26 Range: 8–24	SEEG	Biphasic Pulsewidth: 0.3 ms Current: 9 mA Frequency: 0.9 Hz Trials: 108	CCEP amplitude variation derived using the intertrial standard deviations over the post‐stimulation time course; latency variation derived based on the timing of the first three CCEP deflections	Areas investigated: frontal, temporal, parietal, and occipital regions EZ markers: latency and amplitude variations corrected for Euclidian distance are linked to SPES to and CCEPs measured within the EZ. CCEP variation shows a negative correlation with seizure frequency
Hays 2023^41^	15	Median: 45 Range: 19–54	ECoG, SEEG	Biphasic Pulsewidth: 0.15 ms Current: 4–10 mA Frequency: 0.4/0.5 Hz Trials: 40–50	N1 amplitude	Areas investigated: mesial temporal region EZ markers: Variations in CCEPs across different stimulation intensities may identify epileptogenicity. In the EZ, CCEP responses are maximized at lower current levels, whereas responses outside the EZ progressively increase with higher stimulation intensities
Enatsu 2012^42^	11	Median: 30 Range: 12–52	ECoG, SEEG	Monophasic Pulsewidth: 0.3 ms Current: 1–15 mA Frequency: 1 Hz Trials: 10–54	N1 amplitude and latency	Areas investigated: frontal, temporal, parietal, and occipital regions Contiguous spread is significantly faster than non‐contiguous, but the CCEP distribution is not necessarily correlated with the propagation areas
Enatsu 2012^43^	14	Median: 25 Range: 6–52	ECoG, SEEG	Monophasic Pulsewidth: 0.3 ms Current: 1–15 mA Frequency: 1 Hz Trials: 10–54	N1 amplitude	Areas investigated: frontal, temporal, parietal, and occipital regions EZ markers: N1 amplitude in EZ is significantly larger than in non‐EZ for both paroxysmal fast activity and repetitive spiking electrographic patterns, with amplitudes in repetitive spiking being higher than in paroxysmal fast activity
Iwasaki 2010^44^	10	Mean: 30.2 Range: 9–52	ECoG, SEEG	Monophasic Pulsewidth: 0.3 ms Current: 1–15 mA Frequency: 1 Hz Trials: 10–54	N1 amplitude	Areas investigated: frontal and temporal structures EZ markers: N1 amplitude is significantly higher in the EZ compared to non‐EZ on the group level, suggesting the EZ's increased excitability
Tousseyn 2017^45^	31	Mean: 24 Range: 11–69	SEEG	Biphasic Pulsewidth: 0.3 ms Current: 8 mA Frequency: 1 Hz Trials: 60	RMS power (20–400 ms)	Areas investigated: frontal, temporal, parietal, and occipital regions EZ markers: CCEP powers are significantly larger in hyperperfused areas compared to baseline on SPECT when EZ is stimulated
Shahabi 2021^46^	25	Mean: 29.8 Range: 5–47	SEEG	Not Reported	RMS power (early 10–60 ms, middle 60–250 ms, late 250–600 ms)	Areas investigated: frontal, temporal, parietal, and occipital regions FCD type II displays a more restricted area of hyperexcitability compared to type I, which may involve cortico‐thalamo‐cortical network in FBTCS
Zhang 2018^47^	15	Mean: 21.7 Range: 4–37	SEEG	Biphasic Pulsewidth: 0.3 ms Current: 2–8 mA Frequency: 1 Hz Trials: 50	RMS power (7–300 ms)	Areas investigated: frontal, temporal, parietal, and occipital regions EZ markers: RMS power of CCEP in the EZ is significantly higher than propagation zone in patients with the repetitive spiking electrographic pattern
Kundu 2020^25^	11	Mean: 34.6 Range: 20–50	ECoG, SEEG	Monophasic Pulsewidth: Not reported Current: 2.5–10 mA Frequency: 0.29–0.4 Hz Trials: 20	Mean absolute amplitude (5–100 ms post‐stimulation) and gamma‐band power (70–150 Hz)	Areas investigated: temporal and frontal lobe structures EZ markers: EZ contains 9.9% and 120% larger CCEP amplitude and evoked gamma‐band power, respectively
Guo 2020^48^	25	Mean: 27 Range: 16–47	SEEG	Biphasic Pulsewidth: 0.3 ms Current: 6 mA Frequency: 0.5 Hz Trials: 39–40	Weighted graph using RMS power at 10–300 ms period as edges, N1 amplitudes	EZ markers: the connectivity is highest in EZ and decreases from propagation to non‐involved zones
van ‘t Klooster 2011^49^	13	Mean: 21 Range: 8–42	ECoG	Monophasic Pulsewidth: 1 ms Current: 4–8 mA Frequency: 0.2 Hz Trials: 10	Morlet wavelet transformed time‐frequency features. Frequency bands considered were spike (10–80 Hz), ripple (80–250 Hz), and fast ripple (250–520 Hz)	Areas investigated: frontal, temporal, parietal, and occipital regions EZ markers: evoked fast ripples may be indicative of the EZ, and surgical removal of the corresponding areas may correlate with improved outcomes
Donos 2017^50^	16	Mean: 33.7 Range: 11–47	SEEG	Biphasic Pulsewidth: 3 ms Current: 0.25–5 mA Frequency: 0.07 Hz Trials: 20	Morlet wavelet transformed time‐frequency features at 100–250 Hz, and delayed responses	Areas investigated: frontal, temporal and occipital regions EZ markers: high frequency oscillations and delayed responses have moderate positive predictive values for EZ and may share common generation mechanisms. Combining both biomarkers do not significantly improve EZ localisation
Davis 2018^51^	8	Mean: 25.4 Range: 9–47	ECoG, SEEG	Biphasic Pulsewidth: 1 ms Current: 3.5 mA Frequency: 0.4 Hz Trials: 10	Delayed high‐frequency suppression in the 70–250 Hz frequency range 0.4–1 s after stimulation	Areas investigated: temporal and frontal regions EZ markers: delayed high‐frequency suppression evoked by low‐intensity SPES identified EZ in 6 of 10 patients with a false‐positive rate of 0–0.06
Maliia 2017^52^	20	Mean: 30.7 Range: 9–53	SEEG	Biphasic Pulsewidth: 3 ms Current: 0.25–5 mA Frequency: 0.07 Hz Trials: 20	RMS power of ripple and fast ripple bands during early (10–60 ms) and late (60–500 ms) periods	Areas investigated: frontal, temporal and occipital regions EZ markers: stimulation of the EZ induced a weaker increase in ripple and fast power in the early period, and a stronger inhibition in the delayed periods
Lega 2015^53^	37	Mean: 36 Range: 15–63	SEEG	Biphasic Pulsewidth: 0.3 ms Current: 2–8 mA Frequency: 1 Hz Trials: 30	RMS power in the theta, alpha, beta, low gamma, and high gamma frequency bands	Areas investigated: frontal, temporal, parietal, and occipital regions EZ markers: early spread sites demonstrated greater low gamma band power that is highly coherent with the EZ
Kobayashi 2017^54^	16	Mean: 32.8 Range: 17–55	ECoG	Biphasic Pulsewidth: 0.3 ms Current: 4–12 mA Frequency: 1 Hz Trials: 30	High‐frequency activity powers over the N1 and N2 potentials	Areas investigated: temporal lobe EZ markers: power of high‐frequency activities over N1 potential in the EZ is significantly increased in both ripple and fast ripple bands, particularly in mesial temporal lobe epilepsy
Mouthaan 2016^55^	12	Mean: 19.7 Range: 8–42	ECoG	Monophasic Pulsewidth: 1 ms Current: 8 mA Frequency: 0.2 Hz Trials: 10	Hilbert‐Huang Transformed time‐frequency features of early CCEP responses. Frequency bands considered were spike (10–80 Hz), ripple (80–250 Hz), and fast ripple (250–520 Hz)	Areas investigated: frontal, temporal, and parietal regions EZ markers: early responses (<100 ms) in the ripple band are highly specific for EZ. Early responses are more likely to occur in EZ than propagation
Russo 2023^56^	24	Mean: 43.1 Range: 23–50	SEEG	Biphasic Pulsewidth: 0.5–1 ms Current: 3–5 mA Frequency: 1 Hz Trials: 15	CCEP power from 10 to 150 ms and gamma band activities	Areas investigated: medial temporal lobe and neocortex Seizures triggered by the SPES of medial temporal structures led to an immediate reduction of responsiveness whereas neocortical seizures did not, despite increase in gamma power activity
Kobayashi 2024^57^	12	Median: 26, Range: 18–60	SEEG	Biphasic Pulsewidth: 0.2 ms Current: 4 mA Frequency: 1 Hz Trials: 60	Graph metrics including indegree and outdegree	Areas investigated: temporal and occipital regions Contacts nearest to the responsive neurostimulation sites typically exhibit higher in‐degree CCEPs, which are significantly associated with greater seizure reduction. This indicates that CCEP‐derived connectivity measures could offer valuable insights for optimizing the placement of responsive neurostimulation electrodes
Smith 2022^58^	32	Mean: 33.8 Range: 19–62	ECoG, SEEG	Biphasic Pulsewidth: 0.3 ms Current: 4–10 mA Frequency: 0.5 Hz Trials: 40–50	N1 amplitude and transfer function modeling, which are used to derive Bode plots	Areas investigated: frontal, temporal, parietal, and occipital regions EZ markers: neural resonance, when periodic stimulation at a specific frequency generates high‐amplitude oscillations in the intracranial EEG network that propagate seizure activity
Kamali 2020^59^	22	Mean: 35.0 Range: 18–58	ECoG, SEEG	Biphasic Pulsewidth: 0.3 ms Current: 5 mA Frequency: 0.5 Hz Trials: 50	N1 amplitude and transfer function modeling	Areas investigated: frontal, temporal, parietal, and occipital regions EZ markers: transfer function gains and their associated frequency responses
Hays 2021^60^	18	Median: 33.5 Range: 19–62	ECoG, SEEG	Biphasic Pulsewidth: 0.3 ms Current: 10 mA Frequency: 0.5–1 Hz Trials: 50	N1 amplitude. Graph metrics used were degree centrality, indegree, outdegree, node authority, hub scores, Katz centrality, Katz‐receive, and Katz‐broadcast	Areas investigated: mesial temporal region EZ markers: the epileptogenic mesial temporal region has greater magnitude and density in effective connectivity and outward network centrality
Zhao 2019^61^	8	Mean: 21.5 Range: 13–28	ECoG	Biphasic Pulsewidth: 0.3 Current: Not reported Frequency: 1 Hz Trials: 50	N1 amplitude. Graph metrics used were betweenness centrality, degree centrality, nodal clustering coefficient, nodal efficiency, nodal local efficiency, and nodal shortest path length	Areas investigated: temporal, parietal, and occipital regions EZ markers: EZ tends to have a higher degree centrality and nodal shortest path length than non‐EZ
Parker 2018^62^	7	Mean: 34.6 Range: 26–49	ECoG, SEEG	Biphasic Pulsewidth: 0.5 Current: 4 mA Frequency: 0.2 Hz Trials: 10–40	Absolute amplitude of significant CCEPs at 12–250 ms. Graph metrics used were indegree, outdegree, normalized indegree, normalized outdegree, clustering coefficient, centrality, and reciprocity	Areas investigated: frontal and parietal lobe regions EZ markers: there is a significant increase in CCEP amplitude, outward connectivity and baseline variation in EZ. Increased structural and effective connections from EZ to non‐contiguous areas are also observed
van Blooijs 2018^63^	21	Median: 15 Range: 4–49	ECoG, SEEG	Monophasic Pulsewidth: 1 ms Current: 4–8 mA Frequency: 0.2 Hz Trials: 10	Number of early responses (9–100 ms). Graph metrics used were indegree, outdegree, betweenness centrality, the percentage of bidirectional, receiving and activating connections	Areas investigated: frontal, temporal, parietal, and occipital regions EZ markers: The EZ exhibits increased in‐degree, out‐degree, and bidirectional connectivity but fewer incoming connections, particularly in seizure‐free patients. It is highly self‐connected, with minimal input from non‐EZ regions
Boido 2014^64^	12	Mean: 27.3 Range: 16–39	SEEG	Biphasic Pulsewidth: 2 ms Current: 5 mA Frequency: 1 Hz Trials: 30	Integral areas of N1 and N2 waves. Graph metrics used were density, quality, internal, and external edge parameters	Areas investigated: frontal and temporal regions EZ markers: EZ is characterized by bidirectional connectivity, particularly during the early phases (<60 ms)
Yang 2024^65^	50	Mean: 27.2 Range: 16–47	SEEG	Biphasic Pulsewidth: 0.3 ms Current: 6 mA Frequent: 0.5 Hz Trials: 40	N1 and N2 RMS, N1 onset latency, peak latency, width duration, and area	Areas investigated: frontal, temporal, parietal, and occipital regions The area under the curve for localizing the EZ ranges from 0.88–0.93. N1 RMS is a prominent feature in model decision making
Johnson 2022^66^	10	Mean: 34 Range: 23–51	SEEG	Biphasic Pulsewidth: 0.3 ms Current: 3 mA Frequency: 1 Hz Trials: 10	Randomized subset of 40 channels were chosen for a convolutional neural network	Areas investigated: temporal lobe Deep learning can identify EZ with mean sensitivity of 78.1% and specificity of 74.6%. Delayed responses are more sensitive, whereas early responses are more specific for EZ localization
Malone 2022^67^	7	Not reported	SEEG	Biphasic Pulsewidth: 0.5 ms Current: not reported Frequency: 0.5 Hz Trials: not reported	Extracted features and metadata	Soft ensemble machine learning approaches demonstrated highest precision, recall, and area under the curve in discriminating the EZ
Miller 2021^68^	1	Not reported	ECoG	Biphasic Pulsewidth: 0.2 Current: 6 Frequency: 0.15–0.3 Hz Trials: 10–12	Basis profile curves constructed from principal component analysis	Analyzing the evoked potentials through a summarized basis profile curves in a convergent paradigm allows easy and intuitive interpretation of CCEP data
Miller 2023^69^	2	Mean 41, range 19 to 63	SEEG and scalp EEG	Biphasic Pulsewidth: 0.2 Current: 6 Frequency: 0.15–0.3 Hz Trials: 10	Parametrised canonical response shapes	Parametrisation facilitates the comparison and analysis of variable CCEP data from different brain areas
Norris 2024^70^	35	Median: 17 Range: 4–51	ECoG	Monophasic Pulsewidth: 1 ms Current: 4, 8 mA Frequency: 0.2 Hz Trials: 10	CCEP data within the 9 ms to 1 s post‐stimulation window for classification using transformers	By adopting a convergent approach and considering inter‐trial variability, a transformer model can achieve better classification performance
Wu 2023^71^	8	Range: 19–52	SEEG	Biphasic Pulsewidth: 0.5 Current: 5 mA Frequency: 0.5 Hz Trials: 45	Visual and quantitative features of cortico‐thalamic evoked potentials	Thalamic nuclei involved in epileptic seizure propagation can be identified through visual and quantitative analysis of cortico‐thalamic evoked potentials

*Note*: A range of studies have employed diverse methods to understand CCEPs. Analytical approaches include qualitative assessments, quantitative measurements, spectral analysis, graph‐theoretic methods, and machine learning techniques.

Abbreviations: CCEP, cortico‐cortical evoked potential; ECoG, electrocorticography; EZ, epileptogenic zone; FCD, focal cortical dysplasia; FBTCS, focal to bilateral tonic‐clonic seizures; RMS, root mean square; SEEG, stereo‐electroencephalography; SPECT, single‐photon emission computed tomography; ms, milliseconds; Hz, hertz; mA milliamperes.

Microelectrode recordings have shown that the SPES procedure induces population‐synchronized neuronal firing patterns resembling interictal epileptiform discharges, with similar proportions of neurons showing burst‐only, suppression‐only, or burst‐suppression responses.^72^ This suggests that SPES may activate common cortical mechanisms underlying spontaneous epileptiform activity, thereby providing insights into the network dynamics of epileptic phenomena. Nevertheless, analysis remains challenging due to the large volumes of spatially and temporally complex data. In this article, we examine the methodologies used to analyze CCEPs for EZ localization, characterization of epileptogenic networks, and optimization of surgical outcomes for epilepsy. We discuss both qualitative and quantitative features, spectral analysis, graph‐theoretical metrics, and machine learning techniques aimed at improving the interpretability and clinical utility of CCEP data.

## 3 METHODOLOGY

A literature search was conducted in May 2024 using the PubMed database with keywords such as “cortico‐cortical evoked potential,” “CCEP,” “single‐ pulse electrical stimulation,” “SPES,” “epilepsy,” “epileptogenic,” and “seizure onset.” Studies were included if they involved patients with epilepsy undergoing intracranial SPES via SEEG or ECoG, with subsequent analysis of CCEP data for EZ identification. Full‐text English‐language studies of any design were included. Reviews, conference abstracts, and non‐English publications were excluded. Search results were screened in stages: first by title, then abstract, and finally full text. In addition, reference lists from relevant studies and reviews were screened for any further eligible papers not captured by the initial search.

Data extracted from each paper included author, year of publication, study purpose, number of patients, EZ definition, patient demographics and clinical information, stimulation parameters (including implant type, waveform, pulse width, stimulation current, stimulation frequency, and number of trials), data preprocessing steps, feature extraction methods, analytical techniques, key findings, and study limitations. This extraction process was performed using a piloted proforma.

## 4 QUALITATIVE DESCRIPTORS OF CCEP WAVEFORMS

Studies in the early 2000s primarily used qualitative parameters to analyze CCEP data. In a cohort of 45 patients with drug‐resistant epilepsy (DRE), two types of responses were identified: early (<100 ms) and delayed (100 ms to 1s) responses.^34^, ^35^, ^37^ The early responses were observed consistently in all patients and time‐locked, but the delayed responses were less so. Early responses were characterized by an initial sharp deflection followed by one or two slow waves with alternating polarity. These are considered normal cortical reactions, with variability in polarity, morphology, and latency depending on the individual and the brain regions stimulated. The amplitude of early responses was influenced by stimulation intensity. In contrast, delayed responses, resembling epileptiform discharges, were linked to the EZ in temporal lobe epilepsy and occurred agnostic to the stimulation site. However, the relationship between delayed responses and epileptogenic regions in the frontal lobe was significant but less pronounced than in the temporal lobe, potentially due to the more diffuse nature of seizure onset in frontal regions.^35^ Mechanistically, delayed responses are thought to reflect repetitive reactivation of cortical loops, which may contribute to epilepsy pathophysiology.^34^ Although the morphology of delayed responses remains largely unchanged with variations in stimulus intensity, higher intensities were found to increase their likelihood of occurrence. Indeed, SEEG study with parametric stimulation paradigms examining the dose–response relationship has found the waveforms of pair‐wise CCEPs (5–100 ms post‐stimulation) to have variable magnitude and spatial extent with stimulation up to 5.5 mA.^25^ Beyond this level, the effect is saturated, suggesting an asymptotic threshold for monopolar stimulation.^25^

In patients with frontal‐onset seizures, stimulation of the epileptogenic frontal lobe revealed a distinct late‐response type termed repetitive responses.^35^ These responses, characterized by consecutive sharp‐and‐slow‐wave complexes resembling early responses, were recorded across broader regions, sometimes spanning both hemispheres. They are hypothesized to result from the immediate activation of cortico‐ or thalamo‐cortical loops, triggering neuronal re‐entry. The presence of these markers in EZ suggested their potential as biomarkers to assist in surgical targeting. Indeed, individuals who underwent complete resection of areas showing abnormal CCEP waveforms (i.e., sharp‐and‐slow‐wave complexes) demonstrated significantly better outcomes, with 96% achieving Engel Class I or II status, compared to patients who had only partial (71%) or no (0%) resection.^36^

In the pediatric cohort, these abnormal responses were observed less frequently and inconsistently compared to otherwise similar adult cohorts, even though all patients experienced their typical seizures during monitoring.^37^ This discrepancy may reflect age‐related neurophysiological differences in cortical responses to electrical stimulation, or a consequence of brain networks undergoing plasticity over years in response to the chronic entraining effects of seizures. Notably, axonal conduction delays, estimated by significant CCEPs across brain regions pairs on parcellation data, were found to be ~1.5 ms longer in children younger than 15 years of age compared to older individuals.^23^ Conduction delays along the long‐range fibers appear to be undergo dynamic maturation during adolescence, with conduction latencies gradually shortening into adulthood.^73^ This highlights the potential importance of accounting for age‐related neural development when interpreting CCEP data in pediatric populations.

## 5 TIME‐DOMAIN ESTIMATES OF CCEP WAVEFORMS

Several studies have sought to partially quantify evoked responses by analyzing their amplitude and latency, aiming to overcome the limitations of subjective visual analysis in interpreting electrophysiological data.^74^, ^75^, ^76^ Matsumoto and colleagues considered CCEPs as a two‐part waveform consisting of the initial N1 potential, a first negative deflection peaking around 10–50 ms after the stimulus, followed by the slower N2 potential, which peaks between 50 and 300 ms.^16^, ^77^ By incrementally increasing stimulation intensity, the largest N1 amplitude at maximum current intensity, derived from averaged CCEP responses, was found to be elevated in EZ at a group level.^44^ However, statistically significant differences at the individual level, as opposed to group level, were observed in only three of eight patients. This suggests that heightened cortical excitability in response to low‐frequency electrical stimulation is variable and may be specific to the intrinsic epileptogenicity of each patient.

The N1 amplitude was further examined by segregating patients into two subgroups based on their ictal electrocorticographic features as either paroxysmal fast activity or repetitive spiking.^43^ Regions associated with repetitive spiking exhibited higher N1 amplitudes, suggesting that cortical excitability varies depending on the specific seizure‐onset patterns, which may reflect distinct underlying mechanisms.^78^ Moreover, CCEPs can provide valuable insights into the structural network properties involved in seizure propagation. Although some CCEP‐positive electrodes were associated with ictal propagation, others were not, indicating the presence of connected areas within the EZ that are inhibited from spreading discharges during seizures. This suggests pathological variations in cortical inhibition and excitation thresholds within the epileptic network.^42^ Indeed, intracranial recordings with paired‐pulse inhibition in the mesial temporal lobe revealed enhanced rather than reduced inhibition during interictus.^79^, ^80^ Moreover, evidence from microelectrode arrays shows that the relationship between inhibition and excitation in the epileptic cortex is dynamic, with a critical component being the deterioration of the inhibitory surround.^81^, ^82^ Patients with secondary generalization have demonstrated more electrodes within propagation zones despite negative CCEP responses, indicating the potential role of indirect subcortical propagation in seizure spread.

These earlier studies focused on data obtained from a single maximal stimulation intensity. However, recent research has shown that N1 amplitudes in the EZ increase more sharply but reach their peak at lower stimulation intensities, whereas amplitudes outside the EZ continue to rise with increasing intensity.^41^ This suggests that the EZ is more sensitive to lower stimulation currents, responding below the typical threshold needed to activate physiological network responses. Logistic regression models further confirm that evaluating a range of stimulation intensities improves the accuracy of EZ localization. Furthermore, averaging responses over a stimulation train, as done in previous studies, may mask inter‐trial variability. Notably, the EZ and regions with high interictal spike activity exhibit greater variability across trials, indicated by their higher standard deviations (SDs).^39^ This variability tends to decrease with increasing seizure frequency, likely reflecting the strengthening of neural connections within epileptogenic networks through the synaptic and glial processes associated with seizure occurrence.^40^ Therefore, broader feature spaces may be required to reliably identify epileptogenic activity from CCEP data. Furthermore, many CCEP shapes, particularly with SEEG, do not have a characteristic structure with an N1 component.^69^

## 6 SPECTRAL ANALYSIS OF CCEP RESPONSES

### 6.1 Time‐domain power estimates

**FIGURE 2:**
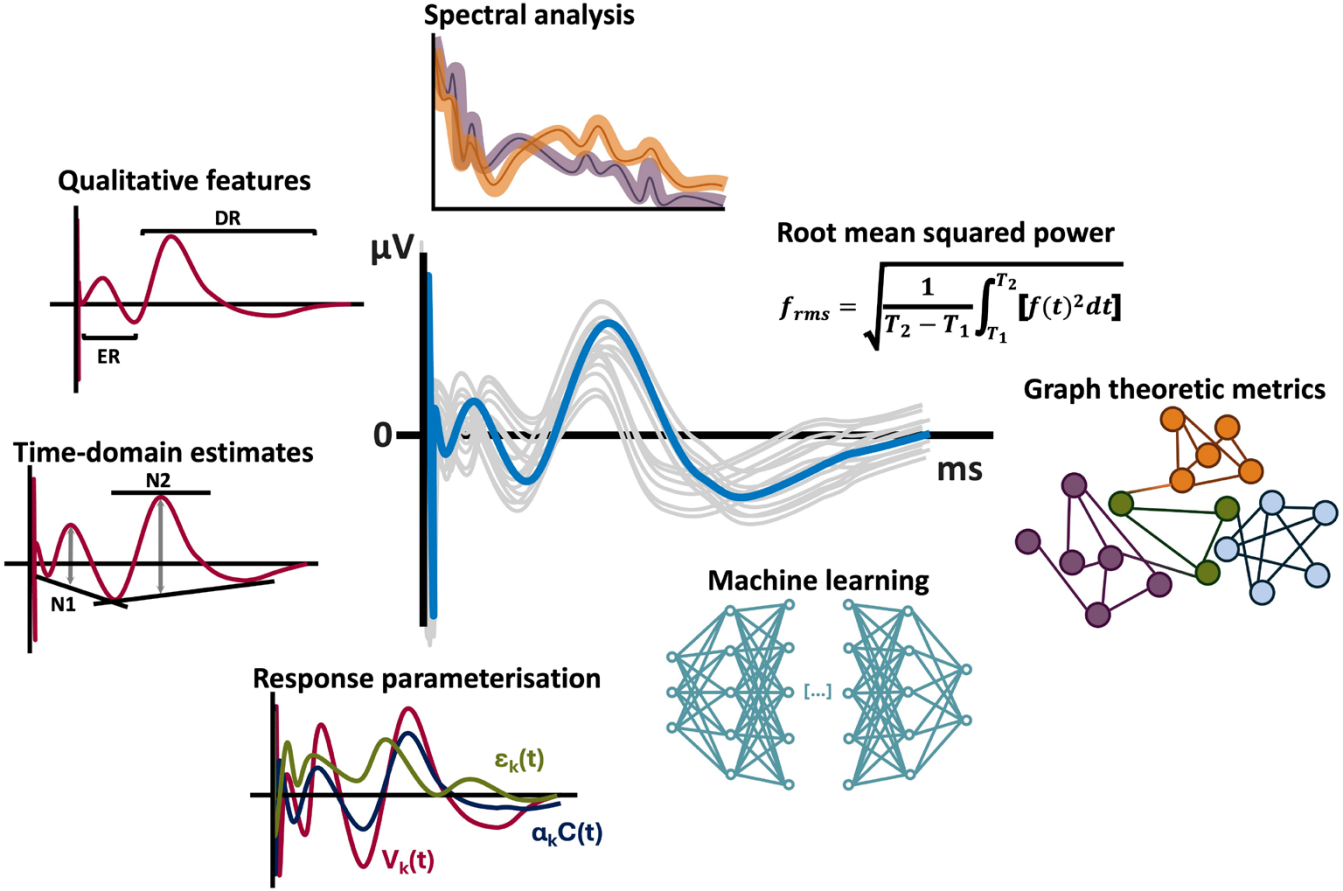
A wide array of techniques has been implemented to interpret CCEP data. Techniques for interpreting CCEP data range from qualitative to advanced analytical methods. Qualitative analyses identify early, delayed, and late waveforms, correlated with physiological or epileptogenic activity. Semi‐quantitative features focus on N1 and N2 components. Spectral analysis detects markers including ripples and fast ripples, and graph‐theoretic metrics highlight the role of EZ as a dense network hub. Response parametrization and machine learning approaches enhance CCEP interpretation by adopting hypothesis‐free approaches.

Some studies have sought to address the challenges of accurately identifying CCEP waveforms by employing power estimations to assess their magnitudes. One such approach is the calculation of root mean square (RMS) (Figure 2). Significant differences in CCEP power have been observed between EZ and non‐EZ from 7 to 300 ms post‐stimulus.^47^ In the repetitive spiking group, CCEP power successfully distinguished between the EZ, non‐EZ, and propagation zones. However, in the paroxysmal fast group, CCEP power was unable to differentiate propagation zones from either the EZ or non‐EZ. These findings align with prior suggestions that distinct seizure‐onset patterns are associated with different underlying electrophysiological mechanisms. In addition, the anatomic proximity between epileptogenic and propagation zones in cases with paroxysmal fast‐onset patterns may partly explain the superior surgical outcomes (Engel Class I) observed in these cases compared to those with repetitive spiking patterns.

By defining specific analysis windows, power estimation provides a valuable framework for investigating the pathophysiology of epilepsy. In a cohort of patients with focal cortical dysplasia (FCD), RMS powers were calculated separately for early (10–60 ms), middle (60–250 ms), and late (250–600 ms) intervals, each corresponding to distinct anatomic pathways thought to be activated.^46^ The CCEP powers observed in the middle and late components, which are hypothesized to relate to the activation of cortico‐thalamo‐cortical pathways, were significantly elevated in FCD type I. In contrast, the early component, thought to reflect cortico‐cortical activation, exhibited greater amplitude in FCD type II, suggesting that type II involves a more localized region of hyperexcitability. These findings may help explain the improved surgical outcomes reported in these patient populations.^83^, ^84^ Nevertheless, the optimal choice of these specific time intervals is not well defined, and the precise demarcation points for intervals of interest currently remain an empirical question.

### 6.2 Time–frequency domain power estimates

Approaches based on time–domain qualitative analysis, amplitude measurements, and power estimation offer simplicity and utility for studying cortical connectivity under electrical stimulation. However, these methods face standardization challenges due to the subjective nature of identifying evoked potentials and the wide variety of descriptive features. CCEP responses in the gamma broadband (70–170 Hz) have been proposed as a physiologically meaningful measure of cortical connectivity, as this signal is well defined in regions with direct anatomic connections to stimulation locations, allowing more precise characterization of causal relationships between brain regions.^85^ These broadband gamma responses demonstrate intuitive relationships with higher stimulation amplitudes producing stronger responses, and increased distance resulting in greater latency. Moreover, these high‐frequency broadband features are particularly advantageous for explicit localization of cortical activations, as they are widely established indicators of average neuronal spiking beneath the electrode.^86^, ^87^, ^88^

Notably, when time–frequency features transformed by the Morlet wavelet were compared with clinically defined EZ, fast ripples (250–520 Hz) emerged as potentially specific markers for epileptogenicity.^49^ Postoperative outcomes showed that patients with a higher percentage of retained fast ripples tended to experience poorer results. However, the sensitivity and positive predictive value of this finding were low. Of interest, the delayed responses previously identified by Valentin and colleagues were hypothesized to share similar generator mechanisms, as they coincided with CCEP high‐frequency oscillations and performed similarly as potential biomarkers.^50^ Nevertheless, the body of literature on CCEP spectral analyses remains unresolved, as conflicting findings have been reported and a heterogeneity of data‐processing pipelines have been implemented. For example, analyses using the Hilbert–Huang transform, which focused specifically on early CCEP responses (<100 ms), found that fast ripple bands, typically considered pathological, were sparse, whereas the ripple band (80–250 Hz) demonstrated high specificity for EZs.^55^ This contradicts earlier suggestions that early responses do not localize epileptogenicity.

Recent studies have examined spectral features using smaller time windows. Ripple and fast ripple power within EZs showed significant increases during the N1 potentials (10–50 ms).^52^, ^54^ In addition, low gamma power (30–70 Hz) was elevated, particularly in regions associated with early ictal propagation, which were defined as the spread of ictal activation within 3 s, indicating dynamic changes in seizure networks during ictal expansion.^53^ This low gamma activity also demonstrated coherence with epileptogenic‐onset sites. In contrast, delayed high‐frequency suppression was found to correlate with EZs, potentially arising from strong inhibition mediated by surrounding γ‐aminobutyric acid (GABA)ergic interneurons.^51^, ^52^, ^54^ This phenomenon may represent the interictal functional disconnection of epileptic foci from adjacent areas, resulting in diminished excitation during the early phase and heightened suppression during the delayed phase.

## 7 NETWORK‐BASED CHARACTERIZATION OF CCEPs

Epilepsy is increasingly recognized as a network disorder, with abnormal interactions between brain regions. Recent research has applied this network framework to the analysis of CCEP data, allowing for richer representations of effective connectivity in the epileptic brain. To construct graphs from CCEP data, cortical regions are represented as nodes, and the connections between them, or edges, are weighted using specific CCEP features such as the N1 amplitude and RMS power. These edge weights may thus map the strength of cortico‐cortical interactions during evoked potentials.

Graph‐theoretic analyses have indicated that the EZ is characterized by an increased nodal shortest path length and average centrality, suggesting denser connectivity with other nodes and a greater capacity for propagating activity within the network.^60^, ^61^ The EZ exhibits heightened outward recruitment and inward responsiveness, with stronger connectivity compared to both propagation zones and non‐EZ regions.^48^ Coupled with their elevated excitability, the EZ functions as unstable network hubs capable of both receiving and transmitting aberrant neural activities. Moreover, the EZ appears to be highly self‐connected, with a greater number of bidirectional connections compared to healthy tissue, featuring dense clusters and numerous internal connections, a hallmark of small‐world network properties.^63^, ^64^ This enhanced interconnectivity with rapid onset and reciprocal activation is thought to involve monosynaptic cortico‐cortical interactions among neuronal groups that occur predominantly during N1 potentials. However, the observed latency remains longer than expected for a pure monosynaptic response (4–8 ms), and stimulation artifacts often saturate clinical amplifiers for 5–10 ms, potentially masking true monosynaptic signals.^22^, ^28^ In addition, transfer functions have been proposed to characterize the relationship between stimulus inputs and epileptic network outputs.^58^, ^59^ It has been suggested that pathological neural resonance aligns with the EZ, and that transfer functions may predict the locations and frequencies of neural oscillations capable of inducing epileptiform activity. However, the broad range of graph theory metrics and methodological variability has introduced challenges in interpreting results and understanding their biological significance, with conflicting findings reported. For example, in a small cohort of patients, epileptogenic tissue displayed increased degree centrality but a reduced percentage of connections from other nodes, whereas betweenness centrality did not reliably indicate epileptogenicity.^63^

Comparisons are made with the structural connectivity profiles derived from probabilistic diffusion tractography, and a pronounced outward connectivity pattern was identified, alongside increased number of non‐contiguous effective connections from epileptogenic to early and late propagation sites.^62^ However, the correlation between effective and reconstructed structural connectivity was found to be low, suggesting possible disease‐induced functional–structural decoupling or fundamental limitations in the current methodology for handling large cortical network data. In addition, CCEP‐based network analysis shows promise for enhancing neuromodulation approaches. SEEG electrodes positioned within 5 mm of eventual responsive neurostimulation (RNS) contacts typically demonstrated larger in‐degree early‐latency CCEPs (10–60 ms), and significant correlation exists between in‐degree CCEPs and greater seizure reduction following RNS.^57^ These findings suggest that CCEPs may aid targeting of pathogenic connectivity and optimize network‐guided neuromodulation.^89^ Endeavors examining the thalamic ictal propagation through cortico‐thalamic–evoked potentials has identified patient‐specific thalamic involvement patterns consistent with seizures of the same type and origin.^71^ It is important to note that the seizure‐onset location does not predict the thalamic nuclei first involved during ictal propagation, suggesting that mapping of personalized subcortical effective connectivity may improve precision neuromodulation approaches.

## 8 MACHINE LEARNING METHODS

Advancements in machine learning have enabled more automated methods for learning data representations and performing classification tasks. These techniques may be well‐suited for analyzing CCEP data due to their ability to handle complex patterns and high‐dimensional, time‐varying data structures. By employing algorithms such as k‐nearest neighbors, and gradient boosting, machine learning successfully integrated multimodal information, including stimulation amplitude, neuroanatomy, tissue type, and laterality, to improve EZ localization.^67^ Several classifiers (logistic regression, stochastic regression/gradient descent, and support vector machines) have shown that N1 power has the greatest influence on localizing EZ compared to other amplitude or latency parameters.^65^

However, conventional machine learning methods require extensive feature extraction and engineering to accurately represent the original data, a task that is further complicated by the inherent variability in CCEP electrophysiological signatures. Several machine learning methods require pre‐analysis, which inherently risks the removal of statistically significant aspects of the CCEP data. Deep learning algorithms; however, require less pre‐analysis and hand‐tuning of the data and have been used to address this concern.^90^ Convolutional neural networks have demonstrated moderate classification performance, achieving a mean sensitivity of 78.1% and specificity of 74.6%.^66^ Further analysis with data segmentation revealed that delayed responses were more sensitive for classifying EZ, whereas early responses were more specific. Transformers, a novel class of deep learning architectures, employ self‐attention mechanisms to assign weights to different channels in the CCEP response.^91^ By incorporating the SD across trials alongside averaged evoked responses, a proposed transformer model was able to account for inter‐trial variability, achieving an area under the receiver‐operating characteristic (ROC) curve (AUC) of 0.745.^70^ Despite these advancements, several promising avenues remain unexplored, such as algorithms that capture temporal dynamics, including gated recurrent units and long short‐term memory networks.^92^, ^93^ Key challenges, including addressing class imbalance and improving model interpretability in epileptic neurophysiological data analysis, remain unresolved.

## 9 TOWARD DATA‐DRIVEN, INTUITIVE CCEP ANALYSIS

A diverse array of analytical techniques has been applied in the literature (Figure 2). However, challenges in the effective analysis of CCEPs persist. Significant variability in CCEP waveforms has been observed consistently, both within and across subjects, complicating the generalization of findings. Factors such as underlying pathology, stimulation parameters, and stimulation sites contribute to the difficulty in identifying a universal, data‐driven electrophysiological biomarker indicative of epileptic activity. Probabilistic analyses indicate that even with standardized data analysis protocols, CCEP results can remain inconsistent. This variability tends to increase with greater distances between stimulation sites and recording electrodes, as well as with lower stimulation intensities.^26^, ^94^ In addition, the choice of implantation type introduces further heterogeneity, as ECoG and SEEG capture distinct network connectivity patterns due to their differing sampling properties.^95^, ^96^, ^97^ Consequently, separate computational preprocessing, including spatial corrections, may be required for depth and subdural electrodes to account for these differences.^83^

Conventional approaches to studying CCEPs typically infer connectivity by analyzing the effects of stimulating a specific site on all other sites. However, this method faces challenges due to variations in the relationship between the underlying cortical laminar architecture and the position of receiving electrodes, making it difficult to consistently interpret CCEP voltage time‐courses within a coherent physiological framework, or distinguishing the different types of interactions.^68^, ^98^ CCEP morphology is known to vary by stimulation location. When stimulation occurs within a single intrinsic network, such as the somatomotor and dorsal attention networks, rapid responses with sharp N1 followed by wider N2 are generated, whereas cross‐network stimulation produces slower responses with N2 only.^99^ The CCEP temporal dynamics follow pathways constrained by intrinsic brain networks. For instance, stimulation of frontoparietal and salience networks triggers fast responses predominantly in salience network locations, whereas default network stimulation generates sustained late responses.^100^ Many previous studies have further constrained their analyses by assuming a canonical temporal structure, such as measuring voltage at fixed delays from stimulation, as seen in N1 and N2 waveforms. However, these assumptions may not universally capture the dynamics of cortical interactions.

In contrast, a “convergent paradigm” that focuses on recording from a single site while stimulating multiple others, may be a more simplified and interpretable approach. By keeping the recording site's anatomy constant, variations in temporal response shapes can reveal fundamentally distinct interactions between cortical regions. Using data‐driven dimensionality reduction techniques CCEPs responses can be characterized using a canonical response shape derived through principal component analysis. This shape is then scaled with a multiplicative factor and a residual term, enabling direct comparisons of diverse trace shapes from different brain areas.^69^ Therefore, the projection strength from each stimulation site can be quantified using concise, standardized metrics without relying on assumptions about specific waveform components or time windows. These simplifications of paradigms to constrain hypotheses about brain networks, complemented by standardized methodology will facilitate more systematic, generalizable, and physiologically insightful investigations of CCEPs in future studies.

## 10 CONCLUSION

Using CCEP, a broad range of analytical techniques has revealed several electrophysiological, spectral, and network characteristics linked to epilepsy. Epileptogenicity may be associated with specific wave morphologies, amplitudes, latencies, high‐frequency oscillations, and graph properties. However, significant methodological variability exists across the literature, with studies differing in demographics, stimulation paradigms, recording locations, and analytical approaches. A deeper understanding of how these factors influence findings, along with the adoption of standardized protocols, is essential to improve the generalizability of CCEP interpretations in identifying epileptogenic foci.

## AUTHOR CONTRIBUTIONS

Cortico‐cortical evoked potentials (CCEPs) are used during intracranial electroencephalography to evaluate effective connectivity between cortical regions, localize epileptogenic zones, and guide epilepsy surgery and neuromodulation approaches. CCEPs produce spatially and temporally complex data, with variability across patients and brain regions, making their interpretation and standardization difficult. Traditional methods focus on qualitative descriptors and semi‐quantitative features including spectral band power and latency‐amplitudes, but they lack consensus on optimal extraction techniques. Emerging solutions including machine learning offer a promising data‐driven approach to enhance biomarker identification and improve the interpretability and clinical utility of CCEP data.

## CONFLICT OF INTEREST STATEMENT

None of the authors has any conflict of interest to disclose. We confirm that we have read the Journal's position on issues involved in ethical publication and affirm that this report is consistent with those guidelines.

## FUNDING INFORMATION

AC is funded by an National Institute for Health and Care Research (NIHR) Academic Clincial Lectureship.

## ETHICS STATEMENT

No new data were collected, and no ethical approval was required.

## Data Availability

No new data were generated or analyzed. All data discussed are available from the original publications cited throughout the text.
